# Association of surgery and economic development in low- and middle-income countries: evidence from a dynamic panel data analysis

**DOI:** 10.1136/bmjgh-2025-021115

**Published:** 2026-07-14

**Authors:** Martilord Ifeanyichi, Johnson Ezema Nchege, Ebikabowei Biedomo Aduku, Meskerem Aleka Kebede, Maeve Bognini, Rachel Hargest, Rocco Friebel

**Affiliations:** 1 Global Surgery Policy Unit, LSE Health, The London School of Economics and Political Science, London, UK; 2 African Centre for Health Economics and Policy Research, Enugu, Nigeria; 3 Department of Economics, University of Nigeria, Nsukka, Enugu State, Nigeria; 4 Department of Economics, University of Africa, Toru-Orua, Bayelsa State, Nigeria; 5 School of Medicine, University Hospital of Wales, Cardiff, UK; 6 Department of Health Policy, The London School of Economics and Political Science, London, UK

**Keywords:** Health economics, Health policies and all other topics, Public Health, Global surgery, LMICs

## Abstract

**Introduction:**

While health improvements drive economic growth, the specific macroeconomic impact of surgery remains unknown. Previous studies have quantified economic losses from unmet need but have not established whether surgery generates economic returns. This study investigates the association between surgical activity and economic growth across low- and middle-income countries (LMICs).

**Methods:**

We analysed panel data from 95 LMICs (2000–2022) using System Generalised Method of Moments (GMM) estimation. Surgical volume was proxied by per capita suture consumption in kilograms (kg) measured by per capita suture imports (in kg), with gross domestic product (GDP) per capita adjusted for purchasing power parity as the outcome variable. We controlled for education, labour force, infrastructure, quality of governance, health expenditure, and credit availability while addressing endogeneity through internal instrumentation.

**Results:**

Each 1% increase in per capita suture consumed corresponded to a 0.0083% increase in per capita GDP (p<0.05). Associations were concentrated and larger in middle-income countries (0.013%; p<0.05) and countries with surgical activity above the 75th percentile in a piecewise spline model (0.016%; p<0.05). No significant associations were found in low-income countries (−0.0039%; p>0.05) or below the 75th percentile surgical activity settings (0.0049%; p>0.05). In a secondary (non-log-transformed) model, a 1-kg increase in per capita sutures consumption corresponded to an increase of 89 international dollars in per capita GDP (p<0.05).

**Conclusion:**

Surgical activity is positively associated with economic growth in LMICs, particularly in higher capacity settings. This provides novel empirical evidence suggesting that surgical investments may yield macroeconomic returns, supporting integration of surgical system strengthening into economic development strategies. Low-income countries may require coordinated investments to build capacity to levels where economic benefits become visible.

**WHAT IS ALREADY KNOWN ON THIS TOPIC**

- Research has demonstrated the contribution of general health improvements to economic development, including gains from increased life expectancy and adult survival rates, but the specific macroeconomic impact of surgical services has not been empirically quantified.

**WHAT THIS STUDY ADDS**

- This is the first study to demonstrate a statistically significant positive association between surgical activity and economic development care in low- and middle-income countries.
- A 1% increase in sutures consumed per capita is associated with a 0.0083% increase in gross domestic product per capita, and this corresponded to an increase of 89 international dollars with a 1-kg increase in sutures consumption per capita in the non-log-transformed model.
- The relationship was stronger in countries with higher surgical activity (0.016%) and in middle-income (0.013%) settings, suggesting that the gains intensify with higher volumes and system readiness.

**HOW THIS STUDY MIGHT AFFECT RESEARCH, PRACTICE OR POLICY**

- Findings reinforce the need to integrate surgical system development into economic planning and public investment agendas, and the absence of association in lower-volume settings signals not a reason to delay investment, but the urgency of scaling surgical systems and building institutional capacity.

## Introduction

 Surgical care remains underdeveloped and underfunded in low- and middle-income countries (LMICs) despite being increasingly recognised as a core component of global health.^1^ In these settings an estimated five billion people lack access to safe, timely and affordable surgical care, leading to avoidable mortality and long-term disability.^2 3^ Studies have implicated several challenges including inadequate infrastructure, a scarcity of trained healthcare professionals and poor motivation of available human resources, shortage of funding, limited administrative capacities, and weak supply systems as barriers to optimum delivery of surgical care.^4^,^7^ Demand side constraints such as lack of awareness about available services and financial costs of accessing care have also been identified.^8 9^ Limited availability and accessibility of surgical care results in a large burden of untreated conditions, contributing to a cycle of poverty and poor health outcomes.^10^ The Global Surgery 2030 report by The Lancet Commission on Global Surgery (LCGS) indicated that the unmet surgical need in LMICs constitute not just a health implication but a significant economic threat, affecting productivity and undermining macroeconomic development,^2^ thus underscoring need for a deeper understanding of the role of surgical care in the broader context of national development.

Economic development, typically measured by indicators such as gross domestic product (GDP) per capita, is a multidimensional indicator that encompasses quantitative and qualitative improvements in the economic and social well-being of a nation’s population.^11 12^ Historically, conversations on economic growth drivers had focused on factors such as industrialisation, infrastructure development and education. However, in recent decades, the focus has expanded to include the role of health in driving economic progress.^13^,^16^

The positive impact of health on a country’s economic development has been established in the literature.^17^ For instance, Bloom and Canning found that a 1-year increase in life expectancy could increase GDP per capita by up to 4%.^13^ The WHO Commission on Macroeconomics and Health also highlighted that investing in health is not merely a social priority but a sound economic strategy.^18^ Using GDP panel data to model the proximate determinants of economic growth at 5-year intervals, Bhargava *et al* reported a consequential 0.05% increase in GDP growth rate with every 1% increase in adult survival rates in poor countries.^14^ Similarly, total life expectancy, male life expectancy and female life expectancy were all found to have positive and statistically significant short-run and long-run effects on both total and per capita income by Neofytidou and Fountas, using a balanced panel of 19 industrial economies and a long time series ranging from 1950 to 2013.^15^ Barro also used a panel of roughly 100 countries observed from 1960 to 1990 in his analysis and concluded that higher life expectancy positively influences GDP growth rate.^16^

Beyond exploring the general health-economic growth nexus, understanding the disaggregated contributions of different health interventions, such as surgery, to economic advancement in LMICs is critical for informed policymaking,^19^ but the specific impact of surgical care on national economic outcomes remains relatively underexplored in the literature. Some recent studies have started to shed light on this area, but most have focused on the economic consequences of the unmet need for surgery due mostly to associated productivity losses. For example, the 2015 LCGS report suggested that without urgent and accelerated investment in surgical scale-up, LMICs will incur an estimated cumulative economic productivity losses amounting to US$12.3 trillion or 1.25% of the GDP between 2015 and 2030.^2 20^ Shrime *et al* reported that about 28% of all disability-adjusted years lost globally were due to surgical conditions.^21^ At a single country level, Adde *et al* reported estimated annual economic losses equivalent to 11–46% of the GDP due to unmet need for surgery in Liberia.^22^ Furthermore, the direct catastrophic and impoverishing impacts of out-of-pocket expenditure in patients seeking surgical care have been established,^23^,^25^ and annual volumes of surgeries performed in a country have been found to correlate with the national expenditure on health.^26 27^ Existing cost-effectiveness studies of specific surgical interventions (eg, caesarean section, cataract surgery) demonstrate substantial returns at the individual level, but these microlevel findings have not been validated at the macroeconomic level.^28 29^

Limited empirical work exists on the direct contribution of surgical operations performed to national macroeconomic development. This study aims to use information from LMICs to provide the first cross-country empirical evidence on the association between surgical activity and national economic development. The findings of this study could contribute to a more nuanced understanding of how investments in surgical care may be associated with economic growth, helping to prioritise surgical care within the broader context of health and economic policy.

## Methods

### Theoretical framework

Broadly, the Solow Growth Model is recognised as the foundational framework for economic growth models^30^; its augmentation by Mankiw, Romer and Weil (1992) explicitly incorporated human capital.^31^ Building on the augmented Solow framework, subsequent studies explicitly incorporated health as a determinant of economic growth and as such healthcare is considered an important contributor to economic development. While the differential nexus between surgical care and economic developmen has not been directly studied, we hypothesise that by the virtue of surgical care being a key production factor of health capital, it logically should contribute to economic development. This position is strengthened by the large body of evidence demonstrating unmet economic potentials due to unmet need for surgery.^2022^,^24^

**Figure 1:**
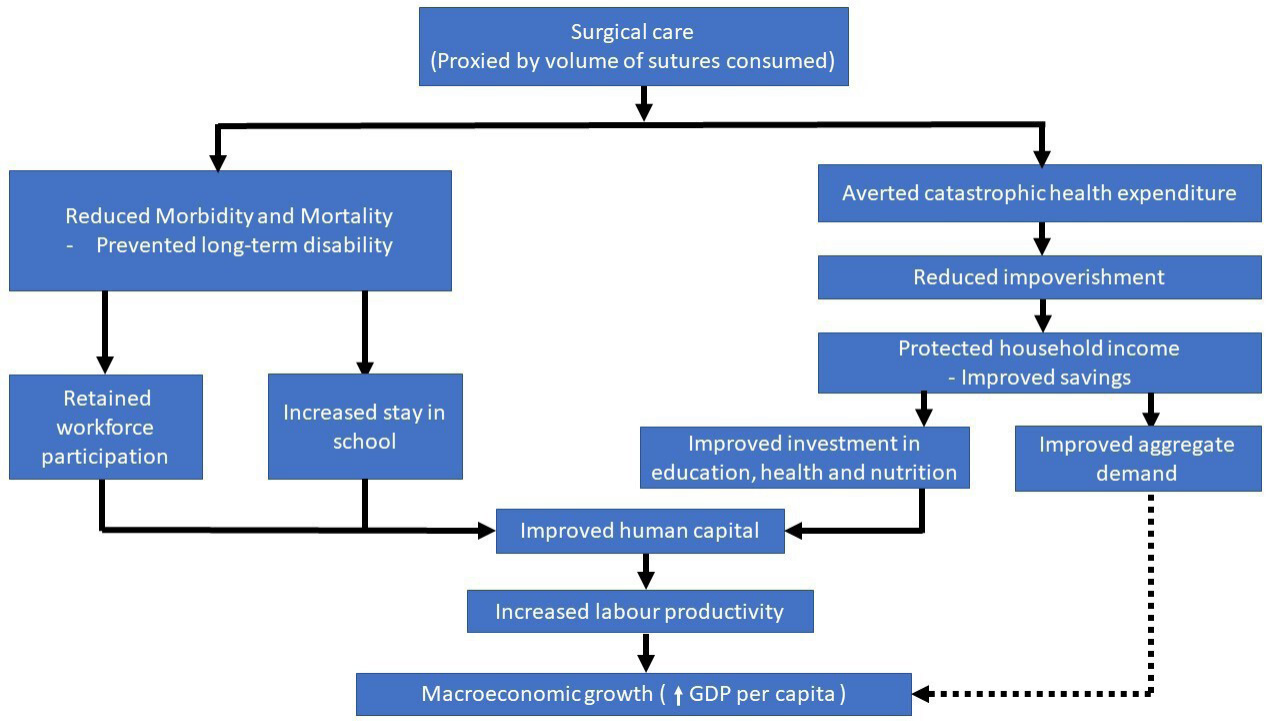
Potential pathways of effect of surgery on economic development. GDP, gross domestic product.

Surgery likely contributes to economic development through multiple interconnected pathways involving improving population health, productivity and financial stability (see figure 1). First, timely and accessible surgical services reduce preventable deaths and long-term disability, enabling individuals to return to work, remain in school and participate fully in economic activities, thereby strengthening the labour force. In addition, effective surgical care lowers household financial burden by preventing catastrophic health expenditures and long-term dependency, which helps preserve savings and supports consumer spending and thus maintains aggregate demand.^2^ Moreover, increased households’ financial capacity enables investments in education, healthcare, nutrition and skill development, which are key determinants of human capital.

### Data and data sources

The data for the study is panel data that spans the years 2000–2022. The measure of economic development used was 2017 constant GDP per capita expressed in purchasing power parity (PPP), to account for cross-country price level differences and facilitate meaningful comparisons across countries. Surgery was expressed in terms of volumes of procedures which were proxied by volumes of sutures in kg per capita imported into the country. Data for volumes of sutures imported were sourced from the United Nations (UN) Comtrade Database.^32^ The data for per capita GDP and other variables of interest, including access to electricity (% of population), government credit to the private sector (% GDP), literacy rates, working age population and government effectiveness were sourced from the World Bank World Development and Governance Indicators.^33^ All countries with more than 25% of the sutures data points missing across the panel were excluded.

The appropriate metric for capturing the intensity or adequacy of surgical activity in a country would be the volumes of surgeries performed in a country relative to the population, for instance, per 100 000 population (ideally disaggregated by procedure types).^2^ However, direct surgical volume data are not available as no comprehensive database systematically captures national panel data on surgical procedures performed in LMICs.^27 34^ We therefore employed volumes of suture imports, which are systematically captured in the UN Comtrade Database as a proxy for volumes of surgeries performed in a country. The original list of study countries comprised all the ‘economies’ (countries+territories) listed as ‘low income’, ‘lower middle income’ or ‘upper middle-income’ by the World Bank in 2023.^35^ For each LMIC, the UN Comtrade was queried using code HS300610 which is specific for surgical sutures. Sutures are a key ingredient in surgical operations and are not amenable to other uses aside from surgical care. Therefore, countries that perform more surgeries are likely to import (and consume) more sutures, creating a positive correlation between our proxy and the construct of interest.

We took several precautions to ensure that suture imports actually represented suture consumption. First, only countries that relied entirely on suture importation were included. Countries that were confirmed or suspected to engage in any degree of local production of sutures (eg*,* Pakistan, India, Brazil and China) were excluded from the analysis. Identification of suture-producing LMICs involved two main complementary mechanisms. First, for each candidate country, we conducted a targeted, in-depth and extensive search of the internet using Google for any record of existence of any sutures manufacturing in the country. This involved the use of combinations of the synonyms of the keywords ‘manufactur*’ + ‘sutures’ + ‘country name’ with deliberate effort to separate actual manufacturers from mere distributors. A country is excluded once a named suture manufacturer/producer is identified in the country, irrespective of the level of local production. Second, the trade data of each country was critically analysed to understand the patterns of suture flows among all LMICs. The UN Comtrade database provides the annual volumes of sutures imported in each country disaggregated by the source countries. As the source countries were mostly the high-income countries for all the countries, appearance of an LMIC as a source/supplier country was easily flagged and recorded. In such cases where an LMIC was listed as a source country, the LMIC was subjected to further targeted Google search and in-country inquiries to determine if the country produces sutures locally, while also critically examining the patterns of sutures inflows into the suspect country. In the absence of any evidence of local suture production or in the case of an isolated supply from a publicly known non-manufacturing import dependent country (eg, Cameroon) to another LMIC (eg, neighbouring Nigeria) such suture flows were assumed to be onward exportation of imported sutures. Furthermore, in such situations where import dependent LMICs exported sutures to other LMICs, the volumes of sutures exported in the year were subtracted from the total amount imported into the country in the year to ensure only the volume internally consumed is captured in the analysis. On the other hand, consistent appearance of an LMIC as a supplier in multiple countries was a ground for suspicion for local production and exclusion. It is noteworthy that we found a clear correlation between the suture trade patterns and Google search findings. Additionally, we reviewed the US Food and Drug Administration database for medical device registrations—which has listings of sutures alongside their manufacturers and their countries of operations (production)—and identified LMICs with active suture manufacturer presence.^36^ By mutual triangulation of the different sources of evidence, we were able to reliably isolate and exclude the sutures producing countries. Although such factors as stock-piling and non-use could disrupt importation-consumption symmetry, we considered that the panel nature of our data and the relatively long lifespan of sutures imply that most if not all the imported sutures are ultimately used. Moreover, the (commercial) vendors that import sutures into the countries are unlikely to import when there is no demand.

Dropping countries with records of local production had an additional benefit of eliminating the potential bias from the direct impact of the associated economic activities of suture manufacturing on the GDP per capita and isolating therefore only the impact of sutures on the GDP per capita through surgery-related health and human capital improvements. This approach also exploits the assumption that imported goods do not directly affect the GDP,^37^ further ensuring that the only link between the imported sutures and GDP is via the pathway of in-country surgery and health and productivity improvements. Meanwhile, asides from the advantage of superior data completeness, this absence of direct reverse impact of imported sutures on GDP per capita distinguishes the use of sutures importation from close potential alternatives such as hospital bed capacity, surgical care provider density, nurse density and physician density.

### Model specification

The augmented Solow growth model is specified as^38^:


(1) $$Y=AK^{a}(hL{)}^{b}$$

Where Y represents the level of output; K represents the physical capital stock, the level of human capital is h and L is labour; and A in equation represents the level of total factor productivity. We adapted this model in our study.

Y is specified as a function of PPP GDP per capita (PCGDP_PPP). The total factor of productivity (A) is influenced by infrastructure, proxied by access to electricity, ELECTRICITY (% of the population), while the stock of physical capital (K) is indirectly represented by government credit to the private sector, CPS (% of GDP), a measure of the capacity to finance capital accumulation, the data of which is more consistently available than the direct measure of the stock of physical capital. The level of human capital (h) is taken as education—proxied by literacy rates (LITR)—and healthcare which is taken as surgery—proxied by the per capita quantity or volume of suture imports (PC_SUTURES). The labour (L), on the other hand, is taken to be the working age population (WORKPOP_PROP)—the proportion of the total population that are within the working age of between 15 years and 64 years. In addition, we control for variations in institutional qualities using government effectiveness (GOVEFF), an approach based on previous work.^39^,^43^ This indicator captures perceptions of the quality of public services, civil service capacity, policy formulation and policy implementation. The scores range between −2.5 and +2.5 with higher values indicating higher effectiveness. Lastly, we include total health expenditure per capita (HEALTH_EXPEND)) as a control variable to account for broader health systems capacities. PC_SUTURES is log-transformed to reduce skewness and mitigate the influence of extreme values. PCGDP_PPP is similarly log-transformed to ensure consistency in functional form and to facilitate ease of interpretation of coefficients (ie, in elasticity terms). To fit a dynamic model and thus capture the state dependence or persistence over time (eg*,* last year’s GDP influencing the current year), we include the first lag of the dependent variable (${PCGDP}_{i,t-1}$) as an independent variable. Thus, the full model specification is given in equation (2) below:


(2) $$\begin{array}{l} log(PCGDP_{it})=b_{0}+b_{1}log(PCGDP_{i,t-1})+\varphi Electricity_{it}\\ +\alpha CPS_{it}+\lambda LITR_{it}+\delta log(PC\_SUTURES_{it})\\ +\vartheta WORKPOP\_PROP_{it}+\beta GOVEFF_{it}\\ +\eta \;HEALTH\_EXPEND_{it}+\gamma_{it}+v_{t}+\varepsilon_{it} \end{array}$$

Where $b_{0}$ is the intercept; $b_{1},\;\varphi ,\;\alpha ,\;\lambda ,\;\delta ,\;\vartheta ,\;\beta ,\;\eta$ are the coefficients; $\varepsilon_{it}$ represents observation-specific errors; $\gamma_{it}$ captures the unobserved country-specific effects; and $v_{t}$ captures the year fixed effects.

### Model estimation

This study employs the System Generalised Method of Moments (GMM) estimator developed by Arellano and Bover^44^ and Blundell and Bond^45^ to estimate the effect of surgical activity on economic development while addressing the key econometric challenge inherent in our model namely endogeneity bias. Endogeneity arises due to the potential bidirectional flow of causality between the GDP per capita (the dependent variable) and sutures consumption and other explanatory variables (reverse causality). For instance, levels of surgical care influence GDP but GDP also influences levels of surgical care.

In the absence of an appropriate external instrument or exogenous variation, systems GMM addresses reverse causality by exploiting internal instruments derived from the panel structure of the data—specifically, lagged values of the endogenous variables (ie, lagged levels in differenced equation and lagged first differences in the level equation). The use of lags is considered effective in breaking the reverse causal link on the assumption that previous volumes of surgical care (eg, last year’s) predict present level of surgical volumes but are not directly influenced by the present GDP per capita. System GMM allows for first-order autocorrelation in the error term but assumes the absence of second-order autocorrelation in the different residuals and this assumption is necessary for the validity of the model moment conditions. We tested for the validity of this assumption using the Arellano-Bond AR(2) test and tested for the validity (exogeneity) of the instruments using the Hansen test.^46^ Further details of the estimation methodology are presented in online supplemental file 1.

### Sensitivity analyses

To ensure the robustness of our findings, we conducted a series of sensitivity checks on our dynamic system GMM model. First, we conducted disaggregated analyses across income groups to assess heterogeneities in association. We assessed non-linearities using a spline model with a knot at the 75th percentile (0.001494 kg). To further explore the presence of a discrete threshold effect, we conducted a grid search over candidate threshold values, defined at the 10th–90th percentiles of the distribution of log-transformed surgical intensity. For each candidate threshold, we estimated a piecewise linear model, and examined whether the slope above the threshold differed from the slope below it. We then compared results across all thresholds to identify a zone where a clear and statistically supported shift in the relationship occurs. Additionally, we modelled access to electricity as an exogenous variable, employed sutures consumption and per capita GDP in levels (not log-transformed), substituted nurse density per 100 000 population for per capita sutures consumption and compared results with an alternative estimation approach, namely ordinary least squares (OLS), to test the consistency of our estimates across methodologies.

### Patient and public involvement

Patients or the public were not involved in the design, conduct, or reporting, or dissemination plans of our research.

## Results

### Descriptive statistics of the variables

Among the 131 countries classified as LMICs by the World Bank in 2023, a total of 95 countries (representing approximately 73%) were included in the study (presented in online supplemental file 2). A total of 36 were excluded for reasons of either local suture production activities (n=24) or data unavailability or deficiency (n=12). Further details are available in online supplemental file 3. Table 1 reports the descriptive statistics of the variables. The mean per capita GDP (PPP-adjusted) was 7453.05 international dollars (I$), with substantial variation (SD=6356.13). Per capita suture imports averaged (mean) 0.0184 kg (approximately 18.4 g per person annually), though with high variability (SD=0.378). The distribution of suture imports was highly skewed, with median imports of 0.00045 kg and 75th percentile of 0.0234 kg, suggesting most countries import relatively small amounts while a few import substantially more. The mean values of working proportion of the population, government effectiveness and access to electricity (% of the population) were 0.60, −0.62 and 63.64%, respectively. Similarly, literacy rates, government credit to private sector and health expenditure per capita averaged 99.76%, 27.34% and US$163.60, respectively.

**Table 1:** Descriptive statistics

Variables	Number of observations	Mean	SD	Minimum value	Maximum value
GDP per capita in PPP (I$)	2137	7453.05	6356.13	621.25	35 688.65
Sutures import per annum per capita (kilograms)	2185	0.018	0.38	0.00	13.68
Government credit to private sector (% of GDP)	1892	27.34	22.27	0.00	180.04
Literacy rates (%)	1888	99.76	19.49	28.29	151.73
Working proportion of the population	2185	0.59	0.08	0.08	0.74
Government effectiveness	2064	−0.6244	0.59	−2.36	1.15
Access to electricity (%) Health expenditure per capita (US$)	2081 2158	63.64 163.60	32.73 196.04	1.28 4.18	100.00 1517.34
T=23 and n=94 where, T is the number of years, and n is the number of panels					

GDP, gross domestic product; I$, international dollar; PPP, purchasing power parity.

### Association of surgery and economic development

The results of the system dynamic panel-data GMM estimation are reported in table 2. The results indicate that per capita suture imports (ie, sutures consumption) have a statistically significant association with GDP per capita, with a coefficient of 0.0083 (p<0.05).

**Table 2:** Results of system dynamic panel-data estimation of surgery effect on per capita GDP PPP

Wald χ² Number of instruments Number of observations Number of groups Average observations per group					18 800 000 (p=0.000) 42 1433 94 15.24	
**Per capita GDP PPP**	**Coefficients**	**Corrected SE**	**Z**	**P value**	**Lower limit (95%**)	**Upper limit (95%**)
Log per capita GDP_t-1_	0.9518726	0.0330831	28.77	0.000	0.8870309	1.016714
Log sutures consumption per capita (kilograms)	0.008303	0.0036599	2.27	0.023	0.0011297	0.0154762
Government effectiveness	0.0099165	0.012706	0.78	0.435	−0.0149868	0.0348197
Working-age population proportion	0.1512385	0.1586485	0.95	0.340	−0.1597068	0.4621839
Literacy rate	0.0000947	0.0010878	0.09	0.931	−0.0020374	0.0022267
Access to electricity	0.0006866	0.000964	0.71	0.476	−0.0012027	0.002576
Government credit to private sector	−0.0002212	0.0003992	−0.55	0.579	−0.0010037	0.0005612
Health expenditure per capita	−0.0000335	0.0000697	−0.48	0.630	−0.0001701	0.000103
Constant	0.3599015	0.2725728	1.32	0.187	−0.1743314	0.8941345
AR(2) Hansen Sargan Difference-in-Hansen					z=−1.52; p=0.128 χ²=10.84; p=0.624 χ²=16.09; p=0.244 χ²=6.02; p=0.422	

GDP, gross domestic product; PPP, purchasing power parity.

This coefficient suggests that a 1% increase in surgical activity is associated with an increase of 0.0083% in GDP per capita, consistent with higher volumes of surgical activities being linked to improved economic outcomes across countries. The lagged dependent variable is also highly significant (p<0.001), underscoring the persistence of economic performance over time. Other covariates—including electricity, government effectiveness, working-age population, literacy rate and credit to the private sector—do not show statistically significant associations with GDP per capita in this model.

Model diagnostics support instrument validity and specification: the Hansen (p=0.624) test does not reject the null of instrument validity, the AR(2) test indicates no second-order serial correlation (p=0.128) and the Difference-in-Hansen test supports the exogeneity of government effectiveness and working population (p=0.422). The Wald χ² statistic (p=0.000) suggests that the explanatory variables in the model jointly have a statistically significant association with GDP per capita.

### Heterogeneity assessment results

The results of the disaggregated analysis by income group are presented in table 3. We find that the positive association between suture consumption and economic development is observed primarily in middle-income countries. In this subgroup, suture imports were significantly associated with higher GDP per capita (PPP) (coefficient=0.013, p<0.05). In contrast, no significant association was observed among low-income countries (coefficient=−0.0039, p>0.05), suggesting a capacity-related gradient.

**Table 3:** Results of the disaggregated analyses by country income groups

	Middle-income countries			Low-income countries		
Wald χ² #Instrument #Observations #Groups Avg observations per group	7 690 000 (p=000) 41 1007 74 13.61			95 100 000 (p=0.000) 32 426 31 13.74		
**Per capita GDP PPP**	**Coefficients**	**Z**	**P value**	**Coefficients**	**Z**	**P value**
Log per capita GDP_t-1_	0.9546207	31.63	0.000	1.02137	12.47	0.000
Log sutures consumption per capita (kilograms)	0.0134709	2.19	0.029	−0.0039326	−0.41	0.681
Government effectiveness	0.0260005	1.05	0.293	0.0192925	0.56	0.578
Work force	0.2991663	1.61	0.107	0.8813354	0.96	0.337
Literacy rate	−0.0045903	−1.85	0.067	−0.0002195	−0.52	0.602
Access to electricity	−0.0005268	−0.38	0.705	−0.0008897	−1.28	0.200
Government credit to private sector	0.0001032	0.17	0.867	−0.0002883	−0.74	0.462
Health expenditure per capita	−0.0000148	−0.31	0.759	−0.0004655	−0.63	0.951
Constant	0.8538154	2.25	0.024	−0.5565767	−1.37	0.170
AR(2) Sargan Hansen Difference-in-Hansen	z=0.17; p=0.868 χ²=10.51; p=0.652 χ²=6.95; p=0.905 χ²=3.67; p=0.721			z=−1.45; p=0.147 χ²=9.63; p=0.022 χ²=1.44; p=0.695 χ²=1.44; p=0.695		

GDP, gross domestic product; PPP, purchasing power parity.

**Figure 2:**
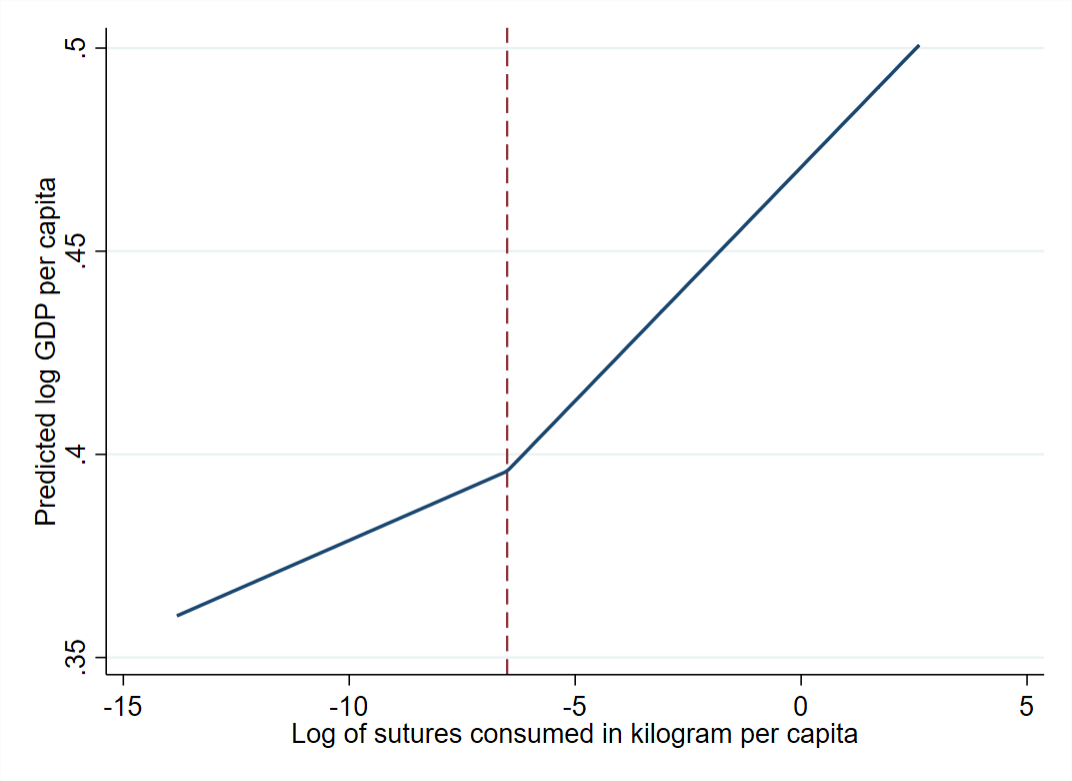
Spline model of the suture—GDP association with knot at 75th percentile. GDP, gross domestic product.

Further, probing of potential heterogeneities through a piecewise spline regression analysis with a threshold imposed at 75th percentile supported the existence of a non-linear relationship, with the slope above the knot being statistically significantly different from the slope below (χ²=7.27; p=0.007). Above the knot, suture imports were positively and significantly associated with GDP per capita, with a larger coefficient of 0.016 (p<0.05), while no significant relationship was observed in the group below the knot (0.0049; p>0.05). These indicate that the slope of the relationship is steeper at high surgical activity percentiles. The full details of the spline results are available in online supplemental file 4. The spline relationship is depicted graphically in figure 2 while the differential marginal effects are illustrated in online supplemental file 5.

The grid search did not identify a statistically significant threshold at any point in the distribution of surgical intensity. The estimated change in slope above candidate thresholds was small and imprecisely estimated across all specifications. However, a consistent pattern emerged: the estimated slope above the threshold increased at higher percentiles of surgical intensity, suggesting a gradual strengthening of the association rather than a discrete structural break. Further details of the grid search results are available in online supplemental file 6.

Instrument validity was generally supported by model diagnostics across all subgroup analyses.

### Robustness check results

The full results of the robustness checks across four model specifications are presented in online supplemental file 7. The results affirmed the core finding suggesting that surgical activity has a positive effect on GDP per capita, although with variations in significance and magnitude. In the model treating access to electricity as exogenous (Model 1), suture imports remained strongly and significantly linked with per capita GDP_PPP (coefficient=0.0087, p<0.05). This relationship held in the levels (non-logged) model (Model 2: coefficient=89.45, p<0.001). In the levels specification with nurse density as the main explanatory variable, a marginally significant positive relationship (coefficient=32.90; p=0.061) was found. In contrast, the OLS model showed a positive but non-significant association (Model 4: coefficient=0.021, p>0.05). Overall, a consistently positive and significant effect was found across all models except OLS, thus reinforcing the potential macroeconomic value of surgical system investments.

## Discussion

This study provides novel cross-country dynamic panel evidence of a positive association between surgical activity and macroeconomic development, extending prior work that has primarily focused on economic losses from unmet surgical need. The finding that a 1% increase in per capita suture consumed is associated with a 0.0083% increase in GDP per capita (I$89 increase for each additional kg of sutures in non-logged model) suggests that surgical care may be an important determinant of economic growth. We find that the relationship is neither linear nor universal, being most evident in middle-income countries and those with higher levels of surgical activity, with progressively increasing elasticities at higher capacity settings. This pattern suggests that economic returns to surgical care become more visible as system capacity and volume rise.

While the estimated average elasticity may appear modest at first glance, its practical significance becomes clearer when considered at scale. A 1% increase in surgical activity corresponds to a 0.0083% increase in GDP per capita, but larger and more policy-relevant changes yield more meaningful economic gains. For example, a 10% increase in surgical activity would be associated with an approximate 0.083% rise in GDP per capita, while a doubling of surgical activity (a 100% increase) corresponds to an increase of roughly 0.83%. In absolute terms, this translates into non-trivial income gains when aggregated across national populations, particularly in middle-income settings where capacities are higher. These findings suggest that even incremental improvements in surgical capacity, when sustained and scaled, may contribute meaningfully to broader economic development. Moreover, nearly twice the average elasticity estimate (0.016%) may be expected under higher capacity scenarios, as revealed in the spline model.

More so, there remain huge opportunities for surgical activity scale up in LMICs. The median sutures import level is only 0.000453 kg per capita; thus, an additional kg for instance represents a movement from near-zero to substantial surgical output, amounting to over 2000-fold surge from the typical levels. While the LCGS recommends 5000 surgical procedures per 100 000 population per year as a minimum threshold for meeting surgical needs, most LMICs average around 877 procedures per 100 000 population and in extreme cases like parts of sub-Saharan Africa rates dip below 150 per 100 000.^47^ This means many countries are thousands of surgeries per 100 000 population away from the benchmark, leaving room for scaled-up surgical activity in thousands of percentages. Large proportional increases in surgical activity are both realistic and necessary, therefore, and the associated economic gains observed in this study may be achievable as countries move toward higher levels of surgical provision.

Comparison with existing literature further contextualises our findings. While Bloom and Canning (2000) found a 4% GDP increase per year of life expectancy gained, our estimate of 0.0083% per 1% increase in sutures per capita consumption suggests surgical care has substantial economic associations relative to its share of health spending. Cost-effectiveness studies of specific interventions provide micro-level support for positive returns, though our macrolevel estimates capture broader spillover effects not visible in individual-level studies.^28 29^

The threshold-like phenomenon with associations concentrated in middle-income countries and higher percentile surgical activity settings deserves careful consideration. We propose that multiple interconnected mechanisms likely contribute to these patterns, operating across supply-side, demand-side and system-level domains. The ”threshold” likely reflects multiple underlying requirements, including infrastructure required for surgical care (eg, electricity, water) that may be absent in the poorest settings, a lack of complementary services essential for surgery (eg, anaesthesia, blood banking and critical care) or demand-side factors such as limited access or affordability of surgical care preventing people from realising the economic benefits.

This framework suggests that low-income countries may require coordinated investments across multiple health system components to reach the level where returns emerge. The absence of associations in low-capacity settings should not be interpreted as evidence that surgery is unimportant in these contexts, but rather that system-level constraints prevent the realisation of economic returns that likely exist at individual and household level.

### Policy implications

The findings have significant implications for health and economic policy in LMICs. First, they provide empirical support for viewing surgical scale-up as an economic investment capable of generating positive effects macroeconomic outcomes.^19^ This perspective may justify increased public financing for surgical infrastructure, workforce development^48^ and supply chains.

Second, the evidence on capacity-related effects underscores that scaling surgical care and enhancing system integration are critical for deriving economic returns from surgical care, requiring therefore a strategic investment approach. While low-capacity settings show limited economic associations, this does not justify disinvestment; on the contrary, it underscores the urgent need for increased, targeted upfront investment to build foundational infrastructure, ensuring countries surpass the critical threshold. Such investments can unlock significant economic benefits, as seen in high-capacity settings, promoting health equity and sustainable growth.

Finally, this study supports the integration of surgical indicators into national development monitoring frameworks. Current global health metrics often overlook surgery despite its cross-cutting relevance to maternal health, injury care, cancer treatment and other major contributors to disease burden.^49^ Recognising and measuring surgery as a potential determinant of economic performance may help elevate its profile in national budget discussions and development assistance.

### Study limitations

Our study has limitations that warrant careful consideration. First, suture imports imperfectly proxy surgical volume. The use of suture consumption as a proxy may be considered innovative in the absence of consistently reported and comparable cross-country data on volumes of surgical procedures in LMICs. However, the relationship between suture use and surgical volume is neither fixed nor uniform: different procedures require varying quantities and types of sutures, and some interventions may use minimal or no sutures at all. As a result, variation in case mix, surgical technique, and supply practices across countries may weaken the correspondence between suture consumption and actual operative volume. This introduces measurement errors that may attenuate or obscure the true relationship between surgical activity and economic outcomes. Moreover, the reporting quality of data to UN Comtrade varies by country and over time, potentially introducing bias to our results. Additionally, we cannot capture informal sector surgical activity or traditional healing practices which may operate in parallel, and this may imply potential underestimation of the effect of surgical care in rural or low-income economies.

Second, there are substantial methodological constraints, most significant of which pertains to residual reverse causality concerns, on account of the lack of truly exogenous variation in surgical volumes. Wealthier countries may import more sutures because they can afford more surgery, and while our lagged specifications and system GMM methodology address this, we cannot claim to have eliminated endogeneity entirely. Moreover, although we control for several potential confounders, we cannot completely rule out omitted variable bias. For example, we lack direct measures of medical tourism flows that might inflate suture imports without proportional domestic economic benefits. Future studies should consider quasi-experimental designs to further validate the relationship between surgical investments and economic growth.

Third, the ecological nature of the data, aggregated at the country level, limits inference to population-level associations and precludes conclusions about individual or facility-level mechanisms linking surgical activity to economic outcomes. Fourth, the use of GDP per capita as the primary outcome for estimating surgical care’s economic associations faces important limitations, especially in low-income settings. For example, temporal mismatches exist between surgical interventions and GDP measurement; GDP also excludes non-market value including household production and quality of life improvements; and therefore, surgical interventions may substantially benefit individual patients and their families without proportionally raising aggregate economic output in low-income countries where informal economic activity dominates. Nonetheless, we note that GDP per capita remains the standard macroeconomic indicator for cross-country comparisons and policy decisions about health system investments.

Fifth, our findings are limited by conceptual challenges in interpretation. Although we frame the relationship in elasticity terms, allowing us to compare changes in predictor and outcome variables in percentages, it remains unclear how many surgeries are represented by 1 kg of sutures and the vast heterogeneity in surgical procedures further complicates interpretation. Future research should explore establishing a clearer mapping between suture usage and surgical volume, and better still, between actual surgical volume disaggregated by procedure type and GDP per capita.

Lastly, by excluding LMICs that produce sutures locally, we strengthened the validity of assumptions that suture imports equal suture consumption, but this limits the generalisability of our findings. Moreover, the identification of countries with local suture production based on internet searches and trade data analyses may be imperfect, potentially resulting in misclassification bias.

### Conclusion

In conclusion, our study helps frame surgery beyond a humanitarian imperative to an economic investment. The capacity-related gradient we document provide a partial explanation for why some countries escape health poverty traps while others remain stuck. For the global development community, our findings suggest that surgical system strengthening deserves integration into core economic development strategies. Future research should focus on identifying optimal investment strategies and the specific capacity thresholds required for economic impact, employing quasi-experimental designs where possible to strengthen causal inference and examining heterogeneity in returns across procedure types and health system configurations. The question is not whether countries can afford investment in surgery, but whether they can afford not to.

## Supplementary material

10.1136/bmjgh-2025-021115 online supplemental file 1

10.1136/bmjgh-2025-021115 online supplemental file 2

10.1136/bmjgh-2025-021115 online supplemental file 3

10.1136/bmjgh-2025-021115 online supplemental file 4

10.1136/bmjgh-2025-021115 online supplemental file 5

10.1136/bmjgh-2025-021115 online supplemental file 6

10.1136/bmjgh-2025-021115 online supplemental file 7

10.1136/bmjgh-2025-021115 online supplemental file 8

## Data Availability

Data are available upon reasonable request.
