# Supplementary material for: Association of surgery and economic development in low- and middle-income countries: evidence from a dynamic panel data analysis
Source: BMJ Glob Health. 2026 Jul 14;11(Suppl 2):e021115. doi: 10.1136/bmjgh-2025-021115 (PMC13374405; doi:10.1136/bmjgh-2025-021115)
Supplement: online supplemental file 1 [file bmjgh-11-Suppl_2-s001.pdf]

Supplementary Material 1: Model Estimation

This study employs the System Generalised Method of Moments (GMM) estimator developed by Arrelano and Bover (1995)(1) and Blundell and Bond (1998)(2) to address key econometric challenges inherent in our model namely endogeneity, autocorrelation, and unobserved heterogeneity. First, endogeneity arises due to the potential the potential bidirectional flow of causality between the GDP per capita and some of the explanatory variables (reverse causality). For instance, volumes of surgical care influences GDP but GDP also influences levels of surgical care. Second, unobserved geographical and demographic time-invariant country characteristics (fixed effects) may be correlated with the explanatory variables thus constituting fixed effects bias. Lastly, the inclusion of the lagged dependent variable ($PGDP_{i,t-j}$) could cause autocorrelation. In this model, volumes of sutures, access to electricity, credit to private sector and literacy rates were treated as endogenous.(3,4) Following standard practice in dynamic panel models, we treat government effectiveness as exogenous, reflecting its institutional inertia and the low likelihood of short-run endogeneity with GDP per capita.(5) Similarly, the size of the working-age proportion is treated as exogenous due to their demographic stability and resistance to short-term shocks in economic output.(6) Lag specification of 1–3 for the lagged dependent variable and 2–3 for the other endogenous regressors was chosen to mitigate simultaneity bias and ensure valid instruments by avoiding contemporaneous correlations with the error term. The inclusion of lag 1 for the lagged dependent variable captures the persistence in GDP dynamics, while deeper lags for the endogenous variables reduce endogeneity risk.(5)

In the absence of an appropriate external instrument or exogenous variation, systems GMM addresses reverse causality by exploiting internal instruments derived from the panel structure of the data – specifically, lagged values of the endogenous variables (i.e., lagged levels in differenced equation and lagged first-differences in the level equation). System GMM is preferred over its precursor and closest alternative, the difference GMM of Arellano and Bond (1991),(7) which relies solely on lagged levels as instruments and is therefore more susceptible to the weak instrument problem - particularly when dealing with highly persistent variables such as $PCGDP_{i,t-j}$, where lagged levels may be only weakly correlated with their first differences.(8,9) In addition, it deals with the unobserved heterogeneity by first-differencing, and allows the inclusion of the time-invariant variables as explanatory variables that would be otherwise ignored in OLS or eliminated in fixed effects or difference GMM estimators, (8,9) and eliminating dynamic-panel associated Nickell bias(10) otherwise seen in fixed effect models.

System GMM allows for first-order autocorrelation in the error term but assumes the absence of second-order autocorrelation in the different residuals and this assumption is necessary for the validity of the model moment conditions. We tested for the validity of this assumption using the Arellano–Bond AR(2) test and tested for the validity (exogeneity) of the instruments using the Hansen test.(11)

Sensitivity analyses

To ensure the robustness of our findings, we conducted a series of sensitivity checks on our dynamic system GMM model. First, we conducted disaggregated analyses across income groups to assess heterogeneities in association. We assessed non-linearities using a spline model with a knot at the 75th percentile (0.001494kg).

To assess whether the relationship between surgical intensity and economic output exhibits a discrete threshold effect, we conducted a grid search over candidate threshold values.(12,13) Thresholds were defined at the 10th to 90th percentiles of the distribution of log-transformed surgical intensity. For each candidate threshold c, we estimated a piecewise linear specification within the system GMM framework, introducing a hinge term defined as:

$$(\text{log\_pc\_sutures} - c)^{+} = \max(0, \text{log\_pc\_sutures} - c)$$

The coefficient on this term captures the change in slope above the candidate threshold. Estimates and standard errors were stored across all candidate thresholds and compared to evaluate the presence of a statistically supported breakpoint.

Additionally, we modelled access to electricity as an exogenous variable, employed sutures consumption and PC GDP in levels (not log-transformed) , substituted nurse density per 100000 population for per capita sutures consumption, and compared results with an alternative estimation approach, namely OLS, to test the consistency of our estimates across methodologies.

References

1. Arellano M, Bover O. Another look at the instrumental variable estimation of error-components models. Journal of Econometrics. 1995 Jul 1;68(1):29–51. doi:10.1016/0304-4076(94)01642-D

2. Blundell R, Bond S. Initial conditions and moment restrictions in dynamic panel data models. Journal of Econometrics. 1998 Nov 1;87(1):115–43. doi:10.1016/S0304-4076(98)00009-8

3. Cracolici MF, Cuffaro M, Nijkamp P. The Measurement of Economic, Social and Environmental Performance of Countries: A Novel Approach. Soc Indic Res. 2010 Jan 1;95(2):339–56. doi:10.1007/s11205-009-9464-3

4. Ogundipe AA, Akinyemi O, Ogundipe OM. Electricity Consumption and Economic Development in Nigeria. International Journal of Energy Economics and Policy. 2016 Jan 18;6(1):1.

5. Roodman D. How to do Xtabond2: An Introduction to Difference and System GMM in Stata. The Stata Journal. 2009 Mar 1;9(1):86–136. doi:10.1177/1536867X0900900106

6. Bloom DE, Canning D. The Health and Wealth of Nations. Science. 2000 Feb 18;287(5456):1207–9. doi:10.1126/science.287.5456.1207

7. Arellano M, Bond S. Some Tests of Specification for Panel Data: Monte Carlo Evidence and an Application to Employment Equations. The Review of Economic Studies. 1991;58(2):277–97.

8. Ali M. Determinants of Related and Unrelated Export Diversification. Economies. 2017;5(4):50. doi:https://doi.org/10.3390/economies5040050

9. Ugbor IK, Ugbor UJ, Aduku E. Governance structure and economic diversification. In. University of Nigeria, Nsukka; 2018.

10. Nickell S. Biases in Dynamic Models with Fixed Effects. Econometrica. 1981;49(6):1417–26. doi:10.2307/1911408

11. Hansen LP. Large sample properties of Generalized Method of Moments Estimators. Econometrica. Econometrica. 1982;50:1029–54.

12. Hansen BE. Threshold effects in non-dynamic panels: Estimation, testing, and inference. Journal of Econometrics. 1999 Dec 1;93(2):345–68. doi:10.1016/S0304-4076(99)00025-1

13. Seo MH, Shin Y. Dynamic panels with threshold effect and endogeneity. Journal of Econometrics. 2016 Dec 1;195(2):169–86. doi:10.1016/j.jeconom.2016.03.005
