# Supplementary material for: Association of surgery and economic development in low- and middle-income countries: evidence from a dynamic panel data analysis
Source: BMJ Glob Health. 2026 Jul 14;11(Suppl 2):e021115. doi: 10.1136/bmjgh-2025-021115 (PMC13374405; doi:10.1136/bmjgh-2025-021115)
Supplement: online supplemental file 2 [file bmjgh-11-Suppl_2-s002.pdf]

Supplementary Material 2: Countries Included in the Analysis with Income Group and World Bank Region

S/N	Country	Income Group	World Bank Region
1	Albania	Upper middle income	Europe & Central Asia
2	Algeria	Upper middle income	Middle East & North Africa
3	Angola	Lower middle income	Sub-Saharan Africa
4	Argentina	Upper middle income	Latin America & Caribbean
5	Armenia	Upper middle income	Europe & Central Asia
6	Azerbaijan	Upper middle income	Europe & Central Asia
7	Bangladesh	Lower middle income	South Asia
8	Belarus	Upper middle income	Europe & Central Asia
9	Belize	Upper middle income	Latin America & Caribbean
10	Benin	Lower middle income	Sub-Saharan Africa
11	Bhutan	Lower middle income	South Asia
12	Bolivia	Lower middle income	Latin America & Caribbean
13	Bosnia and Herzegovina	Upper middle income	Europe & Central Asia

14	Botswana	Upper middle income	Sub-Saharan Africa
15	Burkina Faso	Low income	Sub-Saharan Africa
16	Burundi	Low income	Sub-Saharan Africa
17	Cabo Verde	Upper middle income	Sub-Saharan Africa
18	Cambodia	Lower middle income	East Asia & Pacific
19	Cameroon	Lower middle income	Sub-Saharan Africa
20	Central African Republic	Low income	Sub-Saharan Africa
21	Chad	Low income	Sub-Saharan Africa
22	Comoros	Lower middle income	Sub-Saharan Africa
23	Congo, Dem. Rep.	Low income	Sub-Saharan Africa
24	Congo, Rep.	Lower middle income	Sub-Saharan Africa
25	Costa Rica	High income	Latin America & Caribbean
26	Côte d'Ivoire	Lower middle income	Sub-Saharan Africa
27	Djibouti	Lower middle income	Middle East & North Africa
28	Dominica	Upper middle income	Latin America & Caribbean
29	Dominican Republic	Upper middle income	Latin America & Caribbean
30	Ecuador	Upper middle income	Latin America & Caribbean

31	Equatorial Guinea	Upper middle income	Sub-Saharan Africa
32	Eritrea	Low income	Sub-Saharan Africa
33	Ethiopia	Low income	Sub-Saharan Africa
34	Fiji	Upper middle income	East Asia & Pacific
35	Gabon	Upper middle income	Sub-Saharan Africa
36	Ghana	Lower middle income	Sub-Saharan Africa
37	Grenada	Upper middle income	Latin America & Caribbean
38	Guinea	Lower middle income	Sub-Saharan Africa
39	Guinea-Bissau	Low income	Sub-Saharan Africa
40	Haiti	Lower middle income	Latin America & Caribbean
41	Honduras	Lower middle income	Latin America & Caribbean
42	Indonesia	Upper middle income	East Asia & Pacific
43	Iraq	Upper middle income	Middle East & North Africa
44	Jamaica	Upper middle income	Latin America & Caribbean
45	Kazakhstan	Upper middle income	Europe & Central Asia
46	Kyrgyz Republic	Lower middle income	Europe & Central Asia
47	Lao PDR	Lower middle income	East Asia & Pacific

48	Lebanon	Lower middle income	Middle East & North Africa
49	Lesotho	Lower middle income	Sub-Saharan Africa
50	Liberia	Low income	Sub-Saharan Africa
51	Libya	Upper middle income	Middle East & North Africa
52	Madagascar	Low income	Sub-Saharan Africa
53	Malawi	Low income	Sub-Saharan Africa
54	Maldives	Upper middle income	South Asia
55	Mali	Low income	Sub-Saharan Africa
56	Mauritania	Lower middle income	Sub-Saharan Africa
57	Mauritius	Upper middle income	Sub-Saharan Africa
58	Micronesia, Fed. Sts.	Lower middle income	East Asia & Pacific
59	Moldova	Upper middle income	Europe & Central Asia
60	Mongolia	Upper middle income	East Asia & Pacific
61	Montenegro	Upper middle income	Europe & Central Asia
62	Mozambique	Low income	Sub-Saharan Africa
63	Myanmar	Lower middle income	East Asia & Pacific
64	Namibia	Lower middle income	Sub-Saharan Africa
65	Nepal	Lower middle income	South Asia

66	Nicaragua	Lower middle income	Latin America & Caribbean
67	Niger	Low income	Sub-Saharan Africa
68	Nigeria	Lower middle income	Sub-Saharan Africa
69	North Macedonia	Upper middle income	Europe & Central Asia
70	Papua New Guinea	Lower middle income	East Asia & Pacific
71	Paraguay	Upper middle income	Latin America & Caribbean
72	Rwanda	Low income	Sub-Saharan Africa
73	Samoa	Upper middle income	East Asia & Pacific
74	São Tomé and Principe	Lower middle income	Sub-Saharan Africa
75	Senegal	Lower middle income	Sub-Saharan Africa
76	Serbia	Upper middle income	Europe & Central Asia
77	Sierra Leone	Low income	Sub-Saharan Africa
78	Solomon Islands	Lower middle income	East Asia & Pacific
79	Sri Lanka	Lower middle income	South Asia
80	St. Lucia	Upper middle income	Latin America & Caribbean
81	St. Vincent and the Grenadines	Upper middle income	Latin America & Caribbean
82	Sudan	Low income	Sub-Saharan Africa

83	Suriname	Upper middle income	Latin America & Caribbean
84	Syrian Arab Republic	Low income	Middle East & North Africa
85	Tajikistan	Lower middle income	Europe & Central Asia
86	Tanzania	Lower middle income	Sub-Saharan Africa
87	The Gambia	Low income	Sub-Saharan Africa
88	Timor-Leste	Lower middle income	East Asia & Pacific
89	Togo	Low income	Sub-Saharan Africa
90	Tonga	Upper middle income	East Asia & Pacific
91	Uzbekistan	Lower middle income	Europe & Central Asia
92	Vanuatu	Lower middle income	East Asia & Pacific
93	Yemen, Rep.	Low income	Middle East & North Africa
94	Zambia	Lower middle income	Sub-Saharan Africa
95	Zimbabwe	Lower middle income	Sub-Saharan Africa
