# Supplementary material for: Association of surgery and economic development in low- and middle-income countries: evidence from a dynamic panel data analysis
Source: BMJ Glob Health. 2026 Jul 14;11(Suppl 2):e021115. doi: 10.1136/bmjgh-2025-021115 (PMC13374405; doi:10.1136/bmjgh-2025-021115)
Supplement: online supplemental file 3 [file bmjgh-11-Suppl_2-s003.pdf]

Supplementary Material 3: Low- and Middle-Income Countries Excluded from the Analysis

Table S3a: Countries excluded for local sutures production

S/N	Country	Reason
1	Afghanistan	Evidence of local production
2	Korea, Dem. People’s Rep	Evidence of local production
3	Morocco	Evidence of local production
4	Pakistan	Evidence of local production
5	Egypt, Arab Rep.	Evidence of local production
6	Jordan	Evidence of local production
7	India	Evidence of local production
8	Kenya	Evidence of local production
9	Tunisia	Evidence of local production
10	Ukraine	Evidence of local production
11	Vietnam	Evidence of local production
12	Malaysia	Evidence of local production
13	Mexico	Evidence of local production
14	Brazil	Evidence of local production
15	Bulgaria	Evidence of local production
16	China	Evidence of local production
17	Colombia	Evidence of local production
18	Peru	Evidence of local production
19	Russian Federation	Evidence of local production
20	South Africa	Evidence of local production
21	Thailand	Evidence of local production
22	Türkiye	Evidence of local production
23	Georgia	Evidence of local production
24	Guatemala	Evidence of local production

Table S3b: Countries excluded for data deficiencies or unavailability

S/N	Country	Reason
1	Uganda	Data deficiency
2	Philippines	Data deficiency
3	Iran, Islamic Rep.	Data deficiency
4	West Bank and Gaza	No (sutures) data
5	Kosovo	No (sutures) data
6	Turkmenistan	Data deficiency
7	Palau	Data deficiency
8	Eswatini	Data deficiency
9	South Sudan	Data deficiency
10	Tuvalu	No (sutures) data
11	Somalia	Data deficiency
12	El Salvador	Data deficiency
