# Supplementary material for: Association of surgery and economic development in low- and middle-income countries: evidence from a dynamic panel data analysis
Source: BMJ Glob Health. 2026 Jul 14;11(Suppl 2):e021115. doi: 10.1136/bmjgh-2025-021115 (PMC13374405; doi:10.1136/bmjgh-2025-021115)
Supplement: online supplemental file 4 [file bmjgh-11-Suppl_2-s004.pdf]

Supplementary Material 4: Results of the of the spline model

Table S4: Spline model (75th centile knot)

Wald Chi2	16,000,000 (p = 0.000)
Number of instruments	45
Number of observations	1433
Number of groups	94
Average observations per group	15.24

**Per Capita GDP PPP**	**Coefficients**	**Corrected std err**	**Z**	**p-value**	**Lower limit (95%)**	**Upper limit (95%)**
Log per capita GDP $_{t-1}$	0.9351507	0.0339331	27.56	0.000	0.8686429	1.001658
Sutures import per capita (kilograms): pre-knot	0.004872	0.0041744	1.17	0.243	-0.0033097	0.0130537
Sutures import per capita (kilograms): incremental slope post-knot *	0.0115025	0.0042656	2.70	0.007	0.0031422	0.0198629
Government effectiveness	0.0090954	0.0126017	0.72	0.470	-0.0156035	0.0337944
Working-age population proportion	0.103341	0.1602256	0.64	0.519	-0.2106955	0.4173775
Literacy rate	0.0005007	0.0008387	0.60	0.550	-0.0011431	0.0021445
Access to electricity	0.0013461	0.000932	1.44	0.149	-0.0004807	0.0031729
Government credit to private sector	-0.0003497	0.0003845	-0.91	0.363	-0.0011033	0.000404
Health expenditure per capita	-0.0000208	0.0000669	-0.31	0.755	-0.0001519	0.0001102
Constant	0.4275465	0.2857743	1.50	0.135	-0.1325609	0.9876538
AR(2)	z = -1.57; p = 0.116					

Hansen	Chi2 =19.29; p = 0.201
Sargan	Chi2 = 12.97; p = 0.605
Difference-in-Hansen	Chi2=8.19; p =0.316

* The slope above the knot is the sum of the slope below the knot and the incremental slope above the knot (i.e., 0.0049 + 0.012 = 0.016)
