# Supplementary figures and images for: Association of surgery and economic development in low- and middle-income countries: evidence from a dynamic panel data analysis
Source: BMJ Glob Health. 2026 Jul 14;11(Suppl 2):e021115. doi: 10.1136/bmjgh-2025-021115 (PMC13374405; doi:10.1136/bmjgh-2025-021115)

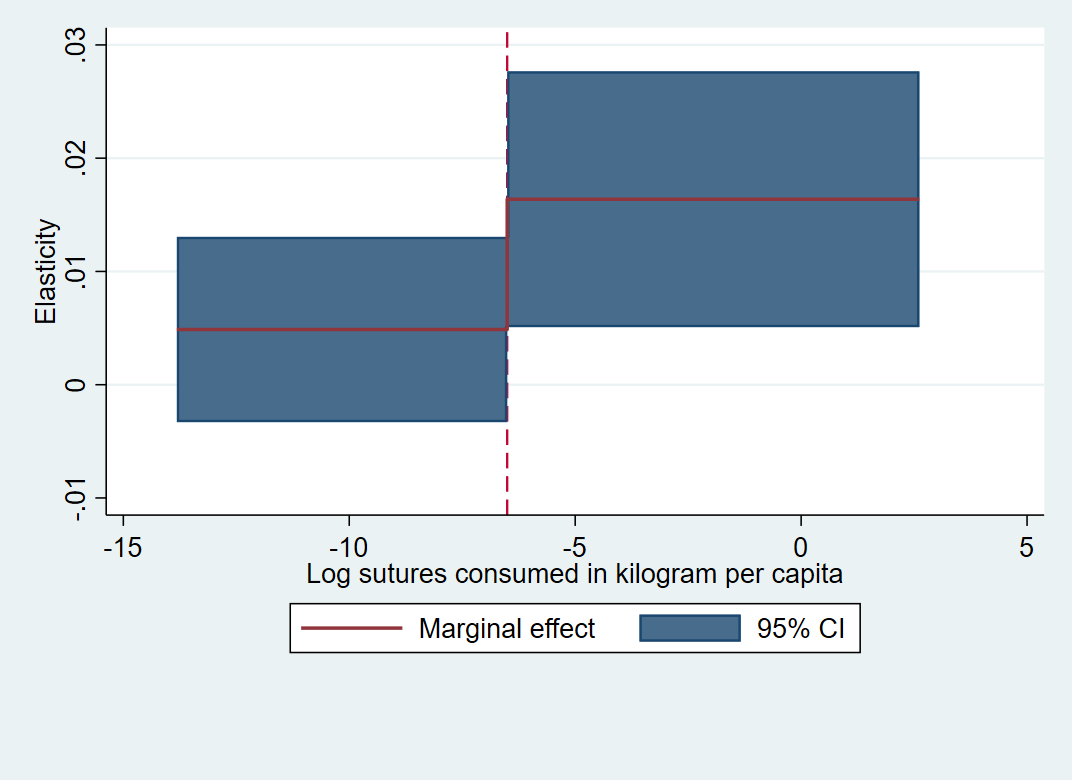

Supplement: online supplemental file 5 [file bmjgh-11-Suppl_2-s005.png]
