# Supplementary material for: Association of surgery and economic development in low- and middle-income countries: evidence from a dynamic panel data analysis
Source: BMJ Glob Health. 2026 Jul 14;11(Suppl 2):e021115. doi: 10.1136/bmjgh-2025-021115 (PMC13374405; doi:10.1136/bmjgh-2025-021115)
Supplement: online supplemental file 6 [file bmjgh-11-Suppl_2-s006.pdf]

Supplementary material 6: Results of threshold grid search for discrete breakpoint

Table S6: Coefficients of the above-knot slope across the range of thresholds

Percentile	Threshold	b_high	SE	t -stat ≈ b/se
10th	-10.39	-0.0036	0.0120	-0.30
20th	-9.28	0.00085	0.0094	0.09
30th	-8.67	0.00214	0.0091	0.23
40th	-8.18	0.00335	0.0089	0.38
50th	-7.70	0.00603	0.0088	0.68
60th	-7.15	0.00760	0.0078	0.98
70th	-6.72	0.00705	0.0067	1.06
80th	-6.19	0.00716	0.0063	1.14
90th	-5.38	0.00737	0.0068	1.09
