# Supplementary material for: Association of surgery and economic development in low- and middle-income countries: evidence from a dynamic panel data analysis
Source: BMJ Glob Health. 2026 Jul 14;11(Suppl 2):e021115. doi: 10.1136/bmjgh-2025-021115 (PMC13374405; doi:10.1136/bmjgh-2025-021115)
Supplement: online supplemental file 7 [file bmjgh-11-Suppl_2-s007.pdf]

Supplementary Material 7: Results of the robustness check analyses

Table S7: Results of the robustness check analyses

Models	(1)	(2)	(3)	(4)
**PCGDP_PPP**	**Coefficients (p value)**			
Per capita GDP $_{t-1}$	0.9337226 (0.000)	0.7698185 (0.000)	.8847919 (0.000)	-
Log sutures consumption per capita (kilograms)	0.0086929 (0.039)	89.44789 (0.000)	-	0.0212288 (0.211)
LogNurse density	-	-	32.89546 (0.061)	-
Government effectiveness	0.0165007 (0.240)	283.5813 (0.409)	11.76438 (0.968)	0.1113196 (0.191)
Work force	0.2143784 (0.356)	503.2477 (0.888)	-2478.074 (0.261)	0.7646152 (0.146)
Literacy rate	-0.0000265 (0.910)	0.2990403 (0.979)	22.8908 (0.179)	-0.0017184 (0.451)
Access to electricity	0.0007977 (0.605)	22.63986 (0.018)	8.485224 (0.437)	0.0186071 (0.000)
Government credit to private sector	-0.0002879 (0.443)	-13.86261 (0.080)	1.919876 (0.811)	-0.0034041 (0.087)
Health expenditure per capita	-0.00000762 (0.917)	4.150139 (0.193)	.9178343 (0.543)	0.0012824 (0.000)
Constant	0.4872766 (0.114)	-187.3311 (0.913)	-1351.39 (0.445)	7.213896 (0.000)
Wald (Chi2, p)<br><br>AR(2) (z, p)<br>Sargan<br>Hansen (Chi2, p)	17,800,000; 0.000<br><br>-1.62; 0.105<br>11.62; 0.309<br>7.91; 0.637	19611.31; (0.000)<br><br>-1.41; 0.159<br>306.07; 0.000<br>20.52; 306.07	30613.39 (0.000)<br><br>-0.27; 0.786<br>55.09; 0.000<br>21.75; 0.243	Not Applicable

Difference-in-Hansen (Chi2, p)	5.46; 0.363	9.67; 0.139	6.09; 0.414	

(1) Modelling access to electricity as exogenous (2) Modelling the levels (non-logged) forms of sutures consumption and PCGDP PPP (3) Substituting sutures consumption with nurse density (4) Pooled OLS estimates.

Abbreviations: OLS, ordinary least squares; PCGDP, per capita gross domestic product; PPP, purchasing power parity.
