# Supplementary material for: Association of surgery and economic development in low- and middle-income countries: evidence from a dynamic panel data analysis
Source: BMJ Glob Health. 2026 Jul 14;11(Suppl 2):e021115. doi: 10.1136/bmjgh-2025-021115 (PMC13374405; doi:10.1136/bmjgh-2025-021115)
Supplement: online supplemental file 8 [file bmjgh-11-Suppl_2-s008.pdf]

BMJ Global Health Author Reflexivity Statement

Adapted from Morton, B., Vercueil, A., Masekela, R., Heinz, E., Reimer, L., Saleh, S., Kalinga, C., Seekles, M., Biccard, B., Chakaya, J., Abimbola, S., Obasi, A. and Oriyo, N. (2022), Consensus statement on measures to promote equitable authorship in the publication of research from international partnerships. Anaesthesia, 77: 264-276. https://doi.org/10.1111/anae.15597

**Study conceptualisation**	
1. How does this study address local research and policy priorities?	An estimated 9 in 10 persons in LMICs lack access to safe, timely and affordable surgery. Results from this study could be potent advocacy tool for strengthening surgical systems in the LMICs
2. How were local researchers involved in study design?	Two LMIC researchers were co-opted from the conceptualisation stage and were involved in the design and execution of the study
**Research management**	
3. How has funding been used to support the local research team(s)?	The study was not funded
**Data acquisition and analysis**	
4. How are research staff who conducted data collection acknowledged?	The research assistant who extracted the data from the relevant database has been recognised in the acknowledgment section.
5. How have members of the research partnership been provided with access to study data?	All the members the research team have access to the data which is freely available in the United Nations Comtrade database
6. How were data used to develop analytical skills within the partnership?	LMIC research partners led and conducted the data analysis phase of the research.
**Data interpretation**	
7. How have research partners collaborated in interpreting study data?	Study results were shared with research partners and they all contributed in interpreting the findings
**Drafting and revising for intellectual content**	
8. How were research partners supported to develop writing skills?	All the research partners were already experienced in writing.
9. How will research products be shared to address local needs?	Research findings will be shared with policymakers and other stakeholders in the forms of journal publication, policy briefs and conference presentations
**Authorship**	

10. How is the leadership, contribution and ownership of this work by LMIC researchers recognised within the authorship?	The two LMIC researchers involved in the project occupy the second and third authorship positions in line with their contributions to the research.
11. How have early career researchers across the partnership been included within the authorship team?	Four out of the total of seven authors are early-career researchers. These include two high-income country-based researchers and the two LMIC researchers.
12. How has gender balance been addressed within the authorship?	The seven authors include four males and three females.
**Training**	
13. How has the project contributed to training of LMIC researchers?	The two LMIC researchers were already experienced in research processes.
**Infrastructure**	
14. How has the project contributed to improvements in local infrastructure?	Not applicable (Study was not funded)
**Governance**	
15. What safeguarding procedures were used to protect local study participants and researchers?	Not applicable (Publicly available dataset was used)
